# Improving lives: Health-related quality of life after surgery for traumatic brachial plexus lesions in a single center retrospective study

**DOI:** 10.1016/j.bas.2026.106136

**Published:** 2026-06-23

**Authors:** Lennart W. Sannwald, Jasmin Greiner, Stefanie Deininger, Benjamin Mayer, Andrej Pala, Andreas Knoll, Christian R. Wirtz, Nadja Grübel, Gregor Antoniadis, Maria T. Pedro

**Affiliations:** a Peripheral Nerve Surgery Unit, Department of Neurosurgery, Ulm University, District Hospital Günzburg, Germany; b Institute of Epidemiology and Medical Biometry, Ulm University, Germany

**Keywords:** Brachial plexus injury, Peripheral nerve surgery, Brachial plexus surgery, Motor outcome, Quality of life, Health-utility

## Abstract

**Introduction:**

Traumatic injuries of the brachial plexus are life-altering, associated with complex injury patterns and commonly affect young people. Since the introduction of brachial plexus microsurgery, studies have largely focused on improving motor outcomes without patient-reported outcome measures.

**Research question:**

To assess the development of quality of life after surgical treatment for traumatic injuries of the brachial plexus in addition to neurological outcome.

**Material and methods:**

This retrospective study investigated neurological outcome and health-related quality of life in 98 patients treated at Ulm University, District Hospital Günzburg between 1st January 2020 and 31st December 2023. EQ-5D-5L-, PainDetect- and two items of the PSQI-questionnaire were sent to all patients regarding their state before surgery and at last follow-up. Clinical data were gathered by chart review.

**Results:**

62 patients returned the questionnaires. Mean follow-up was 22.3 months. MRC grade 2/5 or more was achieved in 59.5% of musculocutaneous nerve, 61.9% of axillary nerve and 54.8% or suprascapular nerve reconstructions. Mean health utility index increased from 0.41 (standard deviation ± 0.34) to 0.57 (±0.28) (p < 0.05) correlating with motor improvement (Spearmans's Rho 0.34, p < 0.05). PainDetect scores showed a significant reduction of mean values from 19.7 (±9.0) to 16.5 (±8.0) (p < 0.05). Sleep duration and quality showed a non-significant trend towards improvement.

**Discussion and conclusion:**

Brachial plexus surgery offers an invaluable possibility to improve patients’ lives and a vital and oftentimes sole therapeutic opportunity for commonly young patients suffering from a profoundly life-altering condition.

## Highlights

• Motor efficiency: strength improvement related to maximum possible improvement.
• EQ-5D-5L, PainDetect and PSQI evaluate quality of life after brachial plexus surgery.
• Quality of life significantly improves after brachial plexus surgery.
• Motor outcome significantly correlates to quality of life improvement.
• Neuropathic pain significantly decreases after brachial plexus surgery.

## 1 Introduction

Introduction of microsurgery in brachial plexus surgery by Millesi and Narakas led to a gradual spread of surgical treatment in the second half of the 20th century and increasing experience with success rates of brachial plexus reconstruction depending on the exact pattern of injury and surgery (Kim et al., 2003; Millesi, 1977; Narakas, 1985). Recovery of shoulder stability, shoulder abduction, elbow flexion against gravity and hand supination gradually emerged as core aims in brachial plexus reconstruction.

However, traumatic injuries of the brachial plexus are commonly associated with high-impact trauma and a wide range of comorbidities in predominantly young people (Karia et al., 2025). Additionally, consequences from brachial plexus injury itself may also manifest in a wide and complex range of symptoms from motor disability to pain and exclusion from everyday life, which makes outcome measurement in brachial plexus surgery particularly challenging (Bengtson et al., 2008). While the amount of case series reporting the neurological outcomes of reconstructive brachial plexus surgery is gradually increasing, knowledge of the impact on patients’ lives remains limited (Dolan et al., 2012; Holdenried et al., 2013; Kretschmer et al., 2009; Rasulic et al., 2017; Choi et al., 1997; Dukan et al., 2023; Christy et al., 2024).

This retrospective study investigates changes in patient-reported outcomes across the domains of health-related quality of life, sleep, pain and employment status following reconstructive brachial plexus surgery. Despite extensive literature on motor recovery of individual surgical techniques, far less is known about how brachial plexus reconstruction affects more general health-related dimensions of patients’ lives (Kim et al., 2003; Millesi, 1977; Narakas, 1985; Birch, 2001; Kandenwein et al., 2005). Measures like muscle strength or electrophysiological reinnervation fall short of reflecting what patients actually experience on a daily basis: long-term emotional, social and functional challenges that often persist long after surgery. However, patient-reported outcomes (PROMs) provide essential insight into quality of life, sleep, pain, and vocational participation. Those domains are increasingly recognized as critical benchmarks of recovery. Yet, data on PROMs after brachial plexus reconstructions remains limited, highlighting the need for long-term studies. The null hypothesis to be falsified is that patients have no significant improvement in health-related quality of life during long term follow-up after reconstructive brachial plexus microsurgery.

## 2 Methods

### 2.1 Study design

The study was designed as a retrospective registry study in order to investigate the neurological outcome and health-related quality of life (QoL) following surgical treatment of traumatic brachial plexus injuries. The study included all patients treated surgically at the nationwide reference center of the peripheral nerve surgery unit of the department of neurosurgery of Ulm University at the District Hospital Günzburg (407 reconstructive nerve surgeries for brachial plexus injuries from 2015 to 2025) between 1st January 2020 and 31st December 2023 due to a traumatic lesion of the brachial plexus independent from pattern of injury or reconstructive surgery. This time window was chosen to enable both sufficient follow-up length and to prevent retrospective quality of life assessment from being influenced too strongly by hindsight. Questionnaires were sent to the resulting study population of 98 patients, 62 of whom eventually responded (response rate 63%). 54 patients (55%) returned all questionnaires completely without missing information.

### 2.2 Principles of surgical treatment

Patients with traumatic brachial plexus lesions are referred to the peripheral nerve surgery unit from across Germany. Upon presentation, a standardized diagnostic work-up is conducted, comprising detailed clinical examination, electrophysiological testing, and radiological imaging to delineate the exact pattern and extent of neural injury. Irrespective of subsequent surgical management, all patients are prescribed structured physiotherapeutic and occupational therapy regimens to facilitate axonal regeneration, maintain joint mobility, and prevent secondary contractures. In the absence of radiological evidence of root avulsion on magnetic resonance neurography (MRN), patients undergo serial clinical and electromyographic assessments at 4–6-week intervals. If no electrophysiological or clinical signs of reinnervation are detectable within the first four to six months post-trauma, operative exploration is indicated within this timeframe. Conversely, in cases where MRN demonstrates definitive root avulsion, early surgical intervention is undertaken. The principal objectives of primary brachial plexus reconstruction consist of restoring glenohumeral stability and achieving reliable elbow flexion, primarily through reconstruction of the suprascapular, axillary, and musculocutaneous nerves. Intraoperatively, injured nerve elements are exposed and subjected to external neurolysis. Subsequently, the intrinsic regenerative potential of the affected nerves is re-evaluated using intraoperative high-resolution ultrasonography, direct electrical stimulation, and nerve action potential recordings (NAPs) (Kline and Happel, 1993; Robert et al., 2009). Based on the number, topography, and gap lengths of the neural structures requiring reconstruction, an individualized reconstructive strategy is formulated. Typically, this involves autologous nerve grafting using sural nerve segments harvested bilaterally; in selected cases, additional graft material from the medial antebrachial cutaneous nerve is employed. Supplementary nerve transfer procedures—such as the Oberlin transfer or spinal accessory-to-suprascapular nerve transfer—are utilized to optimize neurological restitution when indicated. In case of definitive root avulsion the extent of reconstruction has to account for the lack of regenerative capability of the respective myotomes. Remaining intact roots are used directly (root) or indirectly (trunk/cord/nerve) to substitute lost motor function as mentioned above. In the absence of root avulsions, reconstruction focuses on external or internal neurolysis if NAPs can be recorded. If no NAPs are recorded, reconstruction is performed by grafting the intact proximal nerve to nerve sheaths distal to the lesion. Further steps of reconstruction are tailored to the specific lesion pattern and available donor resources. Approximately two years following primary nerve reconstruction, patients undergo secondary evaluation to determine candidacy for muscle or tendon transfer procedures performed by plastic and reconstructive surgeons. Long-term follow-up includes clinical and electrophysiological examinations at least every six months for a minimum duration of two years. Patients are instructed to adhere to intensive physiotherapeutic and occupational therapy protocols—ideally two to three sessions per week—supplemented by daily home-based electrotherapy to maximize functional outcomes.

### 2.3 Collection of clinical data

Patients were included through two different approaches: firstly, patients were informed in writing about the purpose and scope of the registry study. They received an information letter and nine questionnaires. Secondly, to increase the return rate non responding patients were informed about the study details additionally by telephone and asked to participate. Afterwards, they received an online questionnaire.

All questionnaires regarding health-related quality of life were retrospectively answered by patients at last follow-up concerning both the time before reconstructive surgery and at last follow-up. While quality of life data was self-reported retrospectively, motor outcome was prospectively assessed and documented in a specialized outpatient clinic for peripheral nerve surgery preoperatively, immediately after surgery and during follow-up in six month intervals.

The following data was retrospectively collected by chart review: age, sex, lesion characteristics (extent of lesion, mechanism of trauma, presence and pattern of root avulsions), pre- and postoperative strength of the primary target muscles (biceps, deltoid, supraspinatus and infraspinatus according to the grading system of the Medical Research Council (MRC)), mode of surgical treatment (reconstruction or neurolysis, number and types of grafts or transfers, number, length and level of reconstructed nerves).

### 2.4 Patient-reported outcome measures (PROMs)

1. The EQ-5D-5L was used for assessment of health-related quality of life. Patients rate their status in five dimensions (mobility, self-care, usual activities, pain/discomfort, anxiety/depression) on a scale from 1 to 5. Additionally, the current health status is assessed on a visual analogue scale from 0 (worst health) to 100 (perfect health). Based on these responses, a country-specific health utility index and value set is calculated to assess each patient's quality of life (Grochtdreis et al., 2019; Ludwig et al., 2018).
2. PSQI (Pittsburgh Sleep Quality Index): in order to increase the responder rate, two items (sleep duration in hours and sleep quality on a four-point bipolar rating scale) of this validated questionnaire to assess sleep quality over the past four weeks were used (Buysse et al., 1989). These items were chosen to provide a summarizing assessment of core sleep characteristics, as complete PSQI data were not available for all patients. Consequently, no global PSQI score was calculated.
3. The PainDetect questionnaire serves as a screening tool to identify neuropathic pain. It consists of seven questions addressing typical pain characteristics and the pattern of pain progression. The total score ranges from −1 to 38 points: scores ≥19 indicate neuropathic pain, scores between 13 and 18 suggest a possible neuropathic component, and scores ≤12 indicate nociceptive pain (Freynhagen et al., 2006).
4. Additionally, the patients work status at last follow up was registered

The patients completed the questionnaires retrospectively twice in order to assess their condition both preoperatively and postoperatively at the time of data collection.

### 2.5 Statistical analysis

The statistical analysis was conducted using SPSS and Microsoft Excel.

Continuous variables were tested for normal distribution. Metric data were presented as mean values with standard deviation (mean (±standard deviation)), while nominal and ordinal data were reported as absolute and relative frequencies. Due to the heterogeneity of the cohort, medians are additionally presented for the EQ-5D-5L health utility index and PainDetect score.

The primary endpoint of this study is the change in EQ-5D-5L health utility index from preoperative status (retrospectively assessed at last follow-up) to last follow-up. For this, the influence of the independent nominal variable ‘operative status’ (preoperative vs. postoperative at last follow-up) on the dependent ratio-scaled EQ-5D-5L health utility index and PainDetect score were tested for significance using a Wilcoxon-Signed-Rank-test for depending variables.

To quantify and describe motor recovery, a new motor efficiency score was calculated as descriptive individualized measure of motor recovery. This score describes the ratio between the actual improvement achieved and the maximal improvement theoretically possible. It was calculated by division of the postoperative gain in strength (according to the sum MRC of 15 of the deltoid, biceps and supraspinatus muscle) by the potentially possible improvement depending on the preoperative status as follows:

Efficiency = (MRC postOP – MRC preOP) / (15 – MRC preOP)

This efficiency score was calculated only for patients whose preoperative muscle strength was below the maximal value. Exploratory correlation of efficiency with EQ-5D-5L health utility index and postoperative improvement of PainDetect score was evaluated using Spearman's correlation. Due to an alleged crisis of reproducibility, significance testing was restricted to the main hypothesis (Ioannidis, 2005). Regarding this study's sample size and the retrospective design, the explorative significance level was set to p = 0.05 for all analyses.

### 2.6 Ethical principles

The ethics application was approved by the Ethics Committee of Ulm University under application number 448/23. The study was conducted in accordance with the ethical principles of the Declaration of Helsinki in its current version.

## 3 Results

### 3.1 Epidemiological data, pattern of injury and reconstruction

62 patients were included in the study and all quality of life questionnaires regarding the preoperative and postoperative state were completed without missing information in 54 patients. Mean age was 42 years (±18.5, range 16-78, median 37.5) and 52 patients were male (83.9%, 10 female). Mechanisms of trauma were biking accidents in 35 (56.5%) cases, other traffic accidents in 8 (12.9%), skiing accidents in 4 cases (6.5%), iatrogenic injuries in 6 (9.7%) cases and other causes in 9 cases (14.5%). Mean time between trauma and reconstructive surgery was 5.6 (±2.0) months. Root avulsions were diagnosed in 33 (53.2%) patients (Table 1). Reconstructive surgery by graft or nerve transfer was performed in 48 patients (77.4%) (Table 1).

**Table 1:** Number and distribution of root avulsions and nerve reconstructions, including level of reconstruction, number and type of graft and transfer. In two cases a sole reconstruction of one median and one radial nerve was performed, respectively, in addition to neurolysis of the brachial plexus. Overall, sole neurolysis was performed in 14 patients. SD = standard deviation.

Root avulsions 33 patients (100%)	1 root	10 (30.3%)
	2 roots	6 (18.1%)
	3 roots	7 (21.2%)
	4 roots	10 (30.3%)
Affected root levels 82 (100%)	C4	1 (1.2%)
	C5	3 (3.7%)
	C6	18 (22.0%)
	C7	23 (28.0%)
	C8	20 (24.4%)
	Th1	17 (20.7%)
Reconstruction start level	Root	28 (59.6%)
	Trunk	5 (10.6%)
	Cords	2 (4.3%)
	Nerve	12 (25.5%)
Reconstruction end level	Root	1 (2.1%)
	Trunk	6 (12.8%)
	Cords	5 (10.6%)
	Nerve	35 (74.5%)
Reconstructions Musculocutaneous nerve Axillary Nerve Suprascapular nerve	N (%)	Mean reconstruction distance (±SD)
	37 (59.7%)	13.4 cm (±7.5 cm)
	42 (67.7%)	10.4 cm (±4.0 cm)
	31 (50.0%)	6.7 cm (±4.5 cm)

### 3.2 Motor outcome after reconstructive brachial plexus surgery

**Fig. 1:**
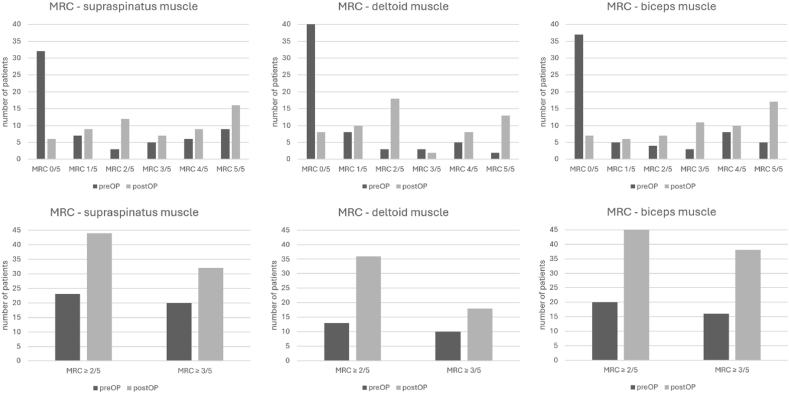
Comparison of pre- and postoperative muscle strength (MRC) across all treated patients (neurolysis and reconstruction combined). Muscle strength grade 2/5 or more of the biceps muscle was achieved in 22 out of 37 cases of musculocutaneous nerve reconstruction (59.5%, 16/37 cases MRC 3 or more), of the deltoid muscle in 26 out of 42 axillary nerve reconstructions (61.9%, 11/42 patients MRC 3 or more), of the supraspinatus muscle in 17 out of 31 cases with suprascapular nerve reconstruction (54.8%, 7/31 MRC 3 or more). MRC 2/5 or more was achieved in 14 out of 14 patients with neurolysis only for biceps muscle strength (13/14 MRC 3/5 or more), 13 out of 14 patients for deltoid muscle strength (11/14 MRC 3/5 or more) and 14 out of 14 patients for supraspinatus muscle strength (14/14 MRC 3/5 or more) at last follow up.

**Fig. 2:**
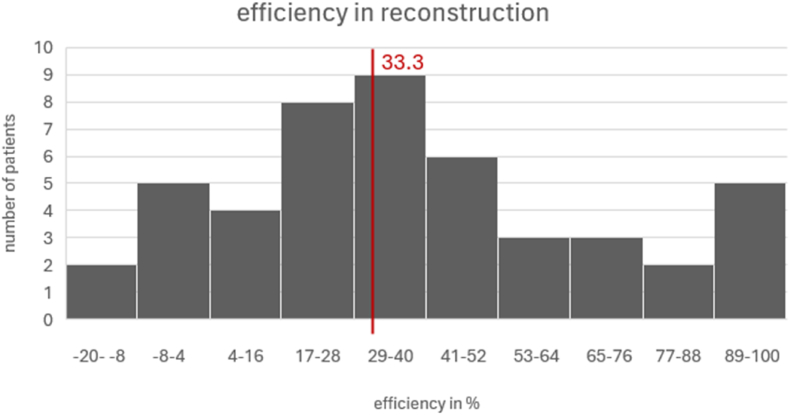
Results of motor efficiency score in surgically reconstructed patients. Calculation of the motor efficiency score is explained in section 2.5. The motor efficiency score was divided in blocks of 12% from −20% to 100%. 100% equals the recovery to the maximum possible strength for the individual patient according to MRC, while 0% equals no motor recovery. Two patients (two in block −20 to −8), which are denoted with negative percentage, showed a postoperative loss of minimal residual motor function without subsequent recovery. All patients in block −8 to 4 showed no motor recovery at all (0% efficiency). The red vertical line represents the median of this cohort's motor efficiency score: 50% of patients show values above and 50% show values below this line.

Mean duration of follow up was 22.3 months (±8.47 months, median 24). Comparison of pre- and postoperative muscle strength of the major target muscles is reported in Fig. 1, while the motor efficiency score is shown in Fig. 2.

### 3.3 Patient reported outcomes regarding health-related quality of life

The collection of subjective data was conducted using standardized questionnaires. The return rate of the completed forms was 63.3% (62 out of 98 patients).

**Fig. 3:**
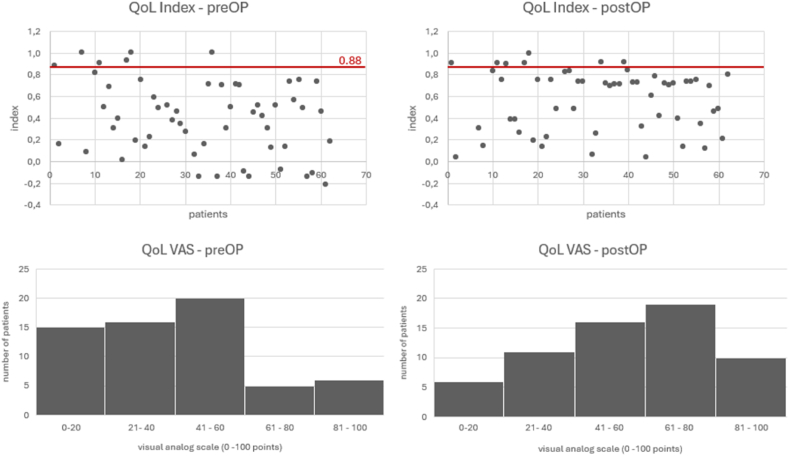
Results of EQ-5D-5L questionnaires pre- and postoperatively. Left column: preoperative, Right column: postoperative, demonstrating a significant shift towards the health utility index of the German population (p < 0.05). Top: Results of the EQ-5D-5L health utility index. The mean index of 0.88 across all age groups in a standardized German population based on a representative sample of 4998 German adults in 2014 is marked by a red line and was chosen due to the age ranging from 16 to 78 in our cohort (Grochtdreis et al., 2019). Bottom: Results of the visual analogue scale for current health status from 0 to 100 grouped in blocks of 20.

The paired scatter plot (Fig. 3) illustrates individual EQ-5D-5L health utility index values before surgery and at last follow-up. Most patients show an upward shift from preoperative to postoperative values with a significant mean health utility index increase from 0.41 (±0.34, median 0.45) preoperatively to 0.57 (±0.28, median 0.72) at last follow-up (p < 0.05). Thus, an overall significant shift of the EQ-5D-5L health utility index of patients with traumatic plexus injury at last follow-up after surgical reconstruction towards values of the German reference population was observed. Moreover, mean values on the visual analogue scale increased from 42.7 (±24.9) preoperatively to 59.4 (±23.6) at last follow-up.

Further exploratory analysis revealed a positive correlation between the motor efficiency score and EQ-5D-5L health utility index postoperatively in patients that received brachial plexus reconstruction (excluding neurolysis only, Spearman's Rho 0.34, p < 0.05). This trend was not found in patients that received neurolysis only (Spearmans's Rho 0.24, p > 0.05).

**Fig. 4:**
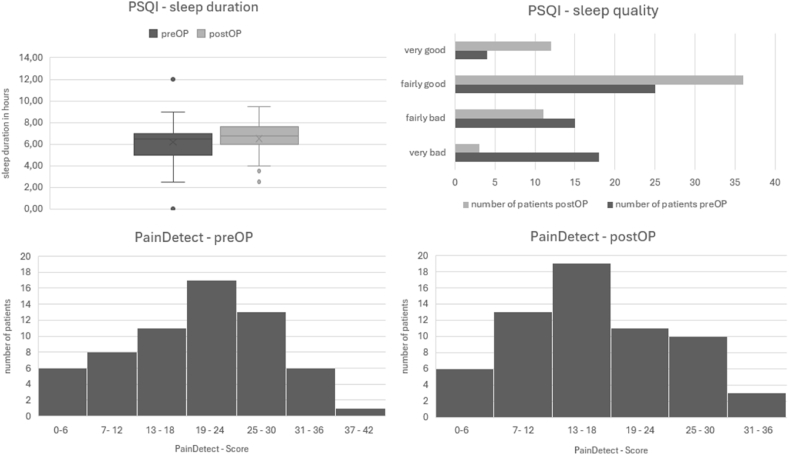
Results of PSQI (top) and PainDetect (bottom) questionnaires. Top: Average sleep duration (left) and quality (right) in the last four weeks before operative (preOP) and at last follow-up (postOP). Bottom: Comparison of PainDetect scores before (preOP, left) and after (postOP) surgery with a significant reduction of mean values from 19.7 to 16.5 crossing the threshold of 18.0 discriminating probable neuropathic pain from indeterminate values (p < 0.05).

Subjective sleep duration before surgery and at last follow-up as inquired by PSQI questionnaire are displayed in Fig. 4. Mean sleep duration changed non-significantly from 6.2 (±2.0) hours preoperatively to 6.5 (±1.4) hours at last follow-up (p > 0.05). However, regarding sleep quality (PSQI question 6) 30 (48.4%) patients reported an improvement of sleep quality of at least one grade (no change in 30 (48.4%) patients, worsening in two patients).

40 patients (64.5%) reported significant pain of probable neuropathic origin (scoring with the PainDetect questionnaire using a final score of 18 or more). However, scoring of neuropathic pain characteristics with the PainDetect questionnaire revealed a significant reduction of mean values from 19.7 (±9.0, median 19.5) preoperatively to 16.5 (±8.0, median 16.0) at last follow-up (p < 0.05, Fig. 4) thus crossing the threshold of 18.0 discriminating probable neuropathic pain from indeterminate values. Overall, out of 62 patients who returned the complete Pain-Detect questionnaire 36 patients (58.0%) reported an improved PainDetect score, while 17 (27.4%) remained stable and 9 (14.6%) patients reported a worse PainDetect score. Exploratory Spearman's correlation revealed no relevant correlation between change in PainDetect score and change in strength as represented by the motor efficiency score (Spearman Rho −0.21, p > 0.05) highlighting the complex genesis of pain in this heterogenous patient cohort. Lastly, 33 (56.9%) patients reported to be employed at last follow-up.

## 4 Discussion

While shoulder stability and elbow flexion remain the traditional primary objectives of nerve surgery for patients with traumatic brachial plexus injury this retrospective study shows that postoperative recovery goes beyond muscle strength alone. Patients reported a significant improvement of EQ-5D-5L derived health utility index values after surgery (mean values 0.41 (±0.34) before surgery and 0.57 (±0.28) at last follow up, p < 0.05). Additionally, a significant postoperative reduction of PainDetect score values was shown, decreasing the mean value below the threshold discriminating probable neuropathic pain from indeterminate values (mean values 19.7 (±9.0) before surgery and 16.5 (±8.0) at last follow up, p < 0.05). Moreover, this study demonstrates a trend towards better sleep quality and an employment rate of 56.9% at last follow up. Lastly, exploratory analysis showed a significant correlation between motor efficiency score and EQ-5D-5L health utility index in patients who received reconstructive surgery (Spearman's Rho 0.34, p < 0.05). Thus, this study is the first to our knowledge to comprehensively investigate health-related quality of life after surgery for brachial plexus injury in a way that enables comparison to a standardized population of normal adults and show a significant improvement and shift towards values in the normal population. While this study shows a significant qualitative shift of health-related quality of life towards the level of the average population (from 0.41 to 0.57, standardized normal population 0.88), it also highlights the relevant and persistent reduction in health-related quality of life these patients experience compared to an average population even after treatment.

Extent of reinnervation corresponds to results reported in the general nerve surgery literature, whereas the variability of functional and neurological recovery directly reflects the heterogeneity of brachial plexus injuries and the biological limits of muscle and peripheral nerve regeneration (Kim et al., 2003; Millesi, 1977; Narakas, 1985; Li et al., 2024). Patients that received neurolysis alone tended to show better neurological improvement than patients receiving reconstructive surgery as a testament to the different biological nature of injury being treated (Millesi et al., 1993).

This reinforces the value of surgery for traumatic brachial plexus lesions which show no spontaneous recovery within three to six months. Kretschmer et al. reported that 87% of patients stated after brachial plexus surgery that they were satisfied with the outcome while 83% of patients would undergo their surgery again (Kretschmer et al., 2009). Similarly, Rasulic et al. reported that 82.6% were satisfied and 81.2% would undergo surgery again (Rasulic et al., 2017). Moreover, Choi et al. stated that 78% of patients with brachial plexus injury in a small cohort reported at least moderate life satisfaction following surgical treatment of their brachial plexus injury (Choi et al., 1997).

Patients with traumatic plexus injuries commonly present with severe trauma after high-velocity accidents and receive complex and long treatment (Karia et al., 2025). The individualized wide range of possible reconstructive surgeries from nerve transfer and transplant to tendon transfer on a plethora of possible nervous and muscular structures makes it difficult to evaluate the effect of brachial plexus surgery on such a holistic outcome measure as quality of life (Bengtson et al., 2008). This study focused on a representative range of well-established patient-reported outcome measures (EQ-5D-5L, PainDetect, PSQI, employment status) in a circumscribed cohort of patients that received brachial plexus surgery with the defined goal of restoring shoulder stability and elbow flexion.

Secondly, neuropathic pain has been reported to be particularly debilitating, severely impacting emotional and social life (Dy et al., 2024). The relevance of neuropathic pain is underscored by a mean PainDetect score above the threshold for probable neuropathic pain, with 64.5% of patients affected. Although the mean score significantly decreased below this threshold at follow-up, no correlation with motor outcomes was observed, highlighting neuropathic pain as an independent challenge. Of note, the PainDetect questionnaire showed limitations, as reduced permanent pain may paradoxically increase scores. While no significant improvement in sleep duration was found, nearly 50% reported better sleep quality. This study provides observational evidence of long-term improvements in health utility, neuropathic pain, sleep quality, and employment alongside neurological recovery. Although not fully reversing injury, reconstructive brachial plexus surgery can restore motor function, reduce pain, and enhance quality of life in a young, socioeconomically relevant population. Further validation through prospective registry studies is needed.

## 5 Limitations

Due to its retrospective design and partial questionnaire return, this study has an inherent and unknown bias towards overrepresenting favorable outcomes. A further bias arises because patients retrospectively assessed both preoperative status and last follow-up at the same time. While patient-reported outcomes are inherently subjective, perceptions vary over time and are influenced by individual circumstances, especially when assessed in hindsight. Nevertheless, subjective wellbeing remains a central goal of care. The design does not allow causal conclusions but only identifies associations in brachial plexus surgery. Limited baseline data for non-responders prevented formal comparison, so selection bias cannot be excluded. Additionally, missing data on pain medication dosage and course restricts full evaluation of postoperative pain development and its influencing factors.

## 6 Conclusion

While shoulder stability and elbow flexion remain primary goals of nerve surgery for traumatic brachial plexus injury, this retrospective study of 62 patients shows recovery extends beyond muscle strength. Patients reported significant improvement in EQ-5D-5L health utility at last follow-up (p < 0.05). PainDetect scores decreased significantly, with mean values falling below the neuropathic pain threshold (p < 0.05). Sleep quality tended to improve, and employment reached 56.9%. A moderate correlation was found between motor efficiency and EQ-5D-5L in reconstructive cases (Spearman's rho 0.34, p < 0.05), but not with PainDetect, highlighting pain complexity. Brachial plexus surgery offers an invaluable possibility to meaningfully improve patients' lives.

## Funding

None.

## Declaration of competing interest

The authors declare that they have no known competing financial interests or personal relationships that could have appeared to influence the work reported in this paper.
